# Cumulative Effect of Cardiovascular Risk Factors on Regulation of AMPK/SIRT1-PGC-1*α*-SIRT3 Pathway in the Human Erectile Tissue

**DOI:** 10.1155/2020/1525949

**Published:** 2020-04-22

**Authors:** Andressa S. Pereira, Alexandra M. Gouveia, Nuno Tomada, Adriana R. Rodrigues, Delminda Neves

**Affiliations:** ^1^Department of Biomedicine-Experimental Biology Unit, Faculty of Medicine of the University of Porto, 4200-319 Porto, Portugal; ^2^Instituto de Investigação e Inovação em Saúde (I3S), 4200-135 Porto, Portugal; ^3^Faculty of Nutrition and Food Sciences, University of Porto, 4200-465 Porto, Portugal; ^4^Department of Urology, Hospital da Luz Arrábida, 4400-346 Vila Nova de Gaia, Portugal

## Abstract

Cardiovascular disease risk factors (CVDRF), especially diabetes mellitus (DM), disrupt oxidative stress response. This condition underlies endothelial dysfunction, early manifested in men as erectile dysfunction. The current study is aimed at elucidating the impact of CVDRF in the oxidation responsive AMPK/SIRT1-PGC-1*α*-SIRT3 pathway and related miRNAs in the human corpus cavernosum. Human penile tissue fragments from individuals submitted to programmed urological surgeries (*n* = 27), aged 43-63 years, were clustered depending on the presence of CVDRF; the control group included samples from patients without CVDRF, and groups A and B included samples from patients with DM and additional CVDRF, totalizing ≤2 CVDRF (group A) and ≥3 CVDRF (group B). Dual-immunolabelling of SIRT3, SOD2, or GPX1 with *α*-actin in tissue sections was carried out. The assessment of expression levels of NOX1, phospho-AMPK*α*, total AMPK*α*, SIRT1, PGC-1*α*, SIRT3, SOD2, and GPX1 was performed by western blotting and of miR-200a, miR-34a, miR-421, and miR-206 by real-time PCR. Phospho-AMPK*α* and SIRT3 expression was found significantly increased in group B relative to other groups, suggesting a marked influence of CVDRF, additional to DM, in the regulation of these enzymes. NOX1 was also increased in group B relative to controls. Only an increasing tendency was observed in the phospho-AMPK*α*/total AMPK*α* ratio, SIRT1, and PGC-1*α* expression in groups A and B when compared with controls. Concerning antioxidant enzymes, GPX1 expression was found incremented in group A, but SOD2 expression was decreased in groups A and B, comparative with controls. Group B presented significantly diminished levels of miR-421 and miR-200a, but only a decreasing trend on miR-34 and miR-206 expression was observed. Taken together, our findings demonstrated that besides DM, additional CVDRF presented a cumulative effect in the cellular response to oxidative unbalance, contributing to AMPK/SIRT1-PGC-1*α*-SIRT3 pathway activation. SOD2, a major mitochondrial antioxidant defence, did not follow the same variation.

## 1. Introduction

Reactive oxygen species (ROS) are generated by multiple reactions within the cell. While in physiological concentrations ROS could be protective or functional, as in the innate immunological response, in unbalanced concentrations, a condition named oxidative stress, ROS provoke cellular toxicity and impairment [1, 2]. Accordingly, increased ROS levels contribute not only to ageing phenotype but also to cardiovascular, neurological, and metabolic diseases and cancer [2–5].

ROS are abundantly formed in the mitochondria, and the enzymes of the family of NADPH oxidases (NOX) were identified as important contributors to their formation [5, 6]. This family includes five NOX (1-5) and two dual oxidase protein (DUOX 1-2) isoforms that present functional and tissue specificity [3, 7]. NOX1, expressed in vascular smooth muscle tissue and endothelial cells, is apparently an important target in diabetes-associated atherosclerosis considering that its inhibition significantly reduces ROS production and vasculopathy [8].

Opposing to ROS formation, detoxifying enzymes, and uncoupling proteins intervene in essential pathways to mitigate oxidative stress [9]. One of such enzymes, mitochondrial superoxide dismutase-2 (SOD2), catalyses superoxide conversion into H_2_O_2_ that is subsequently reduced into innocuous products by enzymes, such as glutathione peroxidase 1 (GPX1), a member of glutathione peroxidases family, located in the cytosol, mitochondria, or even peroxisome [10, 11]. Actually, the modulation of the antioxidant enzymes was found to be crucial for the protection against ROS.

As an upstream regulator of mitochondrial biogenesis and function, the AMP-activated protein kinase (AMPK)/Sirtuin (SIRT)1-peroxisome proliferator-activated receptor *γ* (PPAR*γ*) coactivator-1*α*- (PGC-1*α*-) SIRT3 pathway has been considered an important intervenient in the mitigation of the oxidative stress. Its activation depends on cellular metabolic status. AMPK, a heterotrimer composed of catalytic *α*- and regulatory *β*- and *γ*-subunits, is a nutrient sensor responsive to AMP:ATP ratio increment, conditions of metabolic stress (exercise, glucose deprivation, and hypoxia), and exposure to some hormones and drugs like metformin [12, 13]. The activation of this intracellular serine/threonine protein kinase leads to direct phosphorylation of downstream elements and to regulation of target gene expression, through increment of expression and activation of the transcription factor PGC-1*α* [14, 15]. Interestingly, SIRT1, a mammalian homolog of the yeast Sir2 that belongs to a family of 7 NAD+-dependent histone deacetylases regulates energy metabolism through PGC-1*α* activation [16]. PGC-1*α* modulates the expression of genes involved in energy homeostasis and mitochondria biogenesis through specific regulators, such as estrogen-related receptors (ERRs), PPARs, or nuclear respiratory factors (NRFs) [17]. In addition, Kong et al. demonstrated that PGC-1*α*-ERRs pathway regulates SIRT3 expression and that SIRT3 itself stimulates PGC-1*α*, completing a positive feedback loop [18]. SIRT3 is a mitochondrial matrix enzyme involved in energy homeostasis, mitochondrial biogenesis, and oxidative stress control. Multiple SIRT3 targets have been identified, such as Acetyl-CoA synthetase 2, glutamate dehydrogenase, Ku70, forkhead transcription factor FOXO3a, and antioxidant enzymes [19, 20]. Previous studies demonstrated that ROS production upregulates SIRT3 that further activates SOD2 through deacetylation of the lysine 122 [21–23]. Moreover, SIRT3 was reported to deacetylate FOXO3a and stimulate expression of target genes: SOD2 and GPX1 [19, 20]. SIRT3 plays indeed a major role in downstream pathways of PGC-1*α*, and its impaired function leads to a toxic environment within the cell, potentiating ageing features and multiple diseases. In fact, low levels of SIRT3 were observed in the muscle of mice models of types 1 and 2 diabetes mellitus (DM) [24, 25], in line with data obtained in testes of prediabetic rats that demonstrated decrement of both PGC-1*α* and SIRT3 levels [26]. As well, Freitas et al. found reduced SIRT3 levels in the corpus cavernosum (CC) of aged individuals with metabolic syndrome, supporting the notion that metabolic impairment results in unbalanced ROS levels associated with failure in antioxidant defences [27].

DM, dyslipidaemia, hypercholesterolemia, hypertension, and obesity are cardiovascular disease risk factors (CVDRF), clustered in the metabolic syndrome, long known to be associated with mitochondrial dysregulation, ROS imbalance, and endothelial dysfunction [28]. Endothelial dysfunction, despite being an asymptomatic condition, precipitates atherosclerotic plaques formation and vascular insufficiency that progresses to cardiovascular disease. An early manifestation of endothelial dysfunction in men is erectile dysfunction (ED) [29], which indicates the CC as a proper tissue to study cardiovascular disease-associated molecular modifications.

We thus hypothesize that the AMPK/SIRT1-PGC-1*α*-SIRT3 pathway could suffer disruption in individuals with CVDRF and that modifications could be early detected in the CC. We also consider that not only the elements of this pathway but also microRNAs (miRNAs), small noncoding RNAs that repress expression of target mRNAs, modify its expression and activity with disease. Expression of different miRNAs associated with the regulation of AMPK/SIRT1-PGC-1*α*-SIRT3 pathway has been already identified in the CC of aged or diabetic rodent models and in the CC and serum of nondiabetic patients [30–33]. The SIRT1-PGC-1*α* pathway was reported to be negatively regulated by miR-34a and -200a [33–35] and SIRT3-FOXO3 pathway by miR-421 [36], whereas miR-200a and miR-206 were found to increase in erectile dysfunction (ED) [31].

Herein, we aimed at studying the impact of CVDRF in the AMPK/SIRT1-PGC-1*α*-SIRT3 pathway and in the expression of miRNAs presumably involved in its regulation in the human CC. For this, we used samples collected from individuals with different levels of CVDRF and healthy counterparts, to elucidate if a cumulative effect is present.

## 2. Materials and Methods

### 2.1. Human Penile Tissue Collection and Processing

Samples of CC of penis were obtained from organ donors during organ harvesting for transplant program or, alternatively, from patients submitted to programmed surgeries for correction of penile deviation or implantation of penile prosthesis [37] after informed consent. Patient's age was comprised between 43 and 63 years.

Penile fragments were divided into 3 groups according to the presence, in patients, of CVDRF recognized on the Framingham Heart Study [38], such as DM, dyslipidemia, hypercholesterolemia, hypertension, and obesity. The control group included samples from individuals without ED or CVDRF aged 52.5 ± 5.6 years (*n* = 10). Groups A and B included fragments collected from patients with DM; patients in group A could present an additional CVDRF, on a maximum of two CVDRF (56.6 ± 5.3 years) (*n* = 9), while those included in group B presented two or more CVDRF besides DM (55.4 ± 6.2 years) (*n* = 8).

Briefly, tissue fragments from each patient were excised and divided in two portions; one was immediately frozen at -80°C for molecular analysis and the other fixed in 10% buffered formaldehyde solution and embedded in paraffin for immunofluorescence analysis.

### 2.2. Immunofluorescence

Five *μ*m thick sections of selected samples cut in a microtome (RM 2145, Leica Microsystems GmbH, Wetzlar, Germany) were deparaffinized in xylene and hydrated in a series of aqueous ethanol solutions with decreasing concentration (100%, 90%, and 70% *v*/*v*) followed by water and then submitted to the epitope retrieval in 1 M HCl solution, followed by neutralization with 0.1 M borax solution. Afterwards, sections were incubated with blocking solution (1% *w*/*v* bovine serum albumin (BSA) in phosphate-buffered saline (PBS)) for 1 h and incubated overnight at 4°C in a humidity chamber with a mixture of primary antibodies diluted in the blocking solution: mouse anti-*α*-actin (Millipore, Billerica, MA, USA, 1/400 diluted), combined with rabbit anti-SIRT3 (Cell Signaling Technology, Danvers, MA, USA, 1/200 diluted), anti-SOD2 (Santa Cruz Biotechnology Inc., Paso Robles, CA, USA, 1/250 diluted), or anti-GPX1 (Abcam, Cambridge, UK, 1/600 diluted). After washing, the tissue sections were incubated for 1 h in a humidity chamber with the secondary antibodies anti-mouse conjugated with Alexa Fluor A568 (red) and anti-rabbit conjugated with Alexa Fluor A488 (green) (Molecular Probes, Leiden, Netherlands) both diluted 1/1000 in PBS-0.1% Triton X-100. Sections were mounted in glycerol 50% *v*/*v* in PBS after nuclei staining with 4′-6´-diamino-2-phenylindole (DAPI) (Molecular Probes). Finally, the sections were observed in an ApoTome fluorescence microscope (Imager Z1, Carl Zeiss MicroImaging GmbH, Göttingen, Germany), and the images were acquired with the AxionVision® software (Carl Zeiss MicroImaging GmbH). Representative images of each group were selected. To exclude nonspecific antibody reactivity or autofluorescence, negative controls were prepared without primary or secondary antibodies, respectively.

### 2.3. Western Blotting

Each penile sample was homogenized in lysis buffer (50 mM Tris pH 7.2, 0.1 M NaCl, 5 mM EDTA, 0.5% (*v*/*v*) Triton X-100, and 1 mM *β*-glycerophosphate) supplemented with Protease Inhibitor and Phosphatase Inhibitor Cocktails (Sigma Aldrich Co., Dorset, UK, diluted 0.5% *v*/*v* and 0.2% *v*/*v*, respectively). After quantification of total protein by the method of Bradford [39], 20 *μ*g of protein from each sample was separated by electrophoresis in a 12% SDS-polyacrylamide gel in a Laemmli's discontinuous buffer system (Bio-Rad Laboratories, Inc., Hercules, CA, EUA) during approximately 90 min, at a constant current of 30 mA per gel [40].

Afterwards, proteins were transferred to a nitrocellulose membrane with pore of 0.45 *μ*m (Bio-Rad Laboratories) for 90 min under a constant voltage of 30 V. The image of Ponceau S-labelled protein bands in the membrane was captured in a ChemiDoc TM XRS (Bio-Rad Laboratories, Inc.). The membrane was then washed, incubated with a blocking solution (Tris-buffer saline (TBS) with 0.1% *v*/*v* Tween-20 and 5% *w*/*v* BSA) for 30 min, and then incubated for 48 h with primary rabbit antibodies diluted as indicated: anti-NOX1 1/500 (Santa Cruz Biotechnology Inc.), anti-phospho-AMPK*α* 1/1000 (Cell Signaling Technology), anti-AMPK*α* 1/1000 (Cell Signaling Technology), anti-SIRT1 1/700 (ProteinTech, Chicago, IL, USA), anti-PGC-1*α* 1/500 (Abcam), anti-SIRT3 1/500 (Cell Signaling technology), anti-SOD2 1/1000 (Santa Cruz Biotechnology Inc.), and anti-GPX1 1/1250 (Abcam).

Lastly, several washes and incubation with appropriated secondary antibody coupled to horseradish peroxidase for 1 h were carried out. Labelled bands were detected using chemiluminescent peroxidase substrate (SuperSignal West Pico Chemiluminescent Substrate, Pierce Biotechnology, Rockford, IL, USA), and intensity was quantified with The Image Lab® software (Bio-Rad Laboratories); normalization of protein expression levels was accomplished using Ponceau S staining in the respective lane. The membranes incubated with the anti-phospho-AMPK*α* were further incubated with the rabbit antibody anti-AMPK*α*, after membrane stripping with 10% *w*/*v* SDS for 30 min. The intensity of bands of phosphorylated protein was normalized with the respective total protein band in each penile sample. An *n* = 4 − 6 per group was employed and each experiment was repeated at least three times.

### 2.4. MicroRNA Quantification by Real-Time Polymerase Chain Reaction (PCR)

Three 15 *μ*m thick sections of each paraffin embedded tissue, *n* = 6 − 9 per group, were cut in a microtome (Leica Microsystems GmbH), and total RNA was extracted using the commercial RecoverALL total nucleic acid isolation kit (Ambion, Austin, Texas, USA) according to the method described by Liu and Xu [41]. In brief, sections were deparaffinized with xylene and then washed with 100% ethanol. Afterwards, sections were incubated with a protease solution for 2 h at 50°C, followed by 15 min at 75°C. Total RNA was isolated and DNA enzymatically digested to purify the RNA extract.

The MystiCqTM microRNA cDNA Synthesis Mix (Sigma-Aldrich Co.) was employed to convert RNA into cDNA. The method consists of two steps; in the first one, a mix of 7 *μ*L of RNA sample, 2 *μ*L of poly(A) tailing buffer (5X), and 1 *μ*L of poly(A) polymerase was employed to catalyse the transfer of adenosine deoxynucleotides to the 3′-end of all RNAs, including miRNAs. In the second one, 10 *μ*L poly(A) tailing buffer (5X) and 1 *μ*L of reverse transcriptase (RT) were added to the microRNA cDNA reaction mix for the conversion of RNA into cDNA with incorporation of a unique sequence recognized by a universal primer at the 5′-end of each DNA strand. The amplification of cDNAs by real-time PCR reactions required 1 *μ*L of cDNA from each sample, 0.25 *μ*L of universal primer, 1.2 *μ*L of specific primer, 6 *μ*L of PowerUp™ SYBRTM Green Master Mix (Invitrogen, Carlsbad, CA, EUA), and 3.55 *μ*L of nuclease-free water. Specific primers (RNx) for miR-200a, miR-34a, miR-421, and miR-206 (Qiagen, Hilden, Germany) were employed. Two control reactions were performed, one excluded poly(A) polymerase and the other excluded RT. Reactions were prepared in duplicate in 96-well thin-wall PCR plates and took place in a StepOnePlus™ Real-Time PCR system (Life Technologies, CA, USA), with amplification conditions starting with 95°C for 10 min, followed by 45 cycles of 95°C for 15 s, 55°C for 30 s, and 60°C for 30 s. Finally, the data from each amplification reaction was normalized with the internal control RNU1A, using the RNU1A primer assay (Qiagen) and the formula 2^(CT RNU1A–CT RNx)^.

### 2.5. Statistical Analysis

The results are presented as mean ± standard error of the mean (SEM). Statistical analysis was performed by one-way analysis of variance (ANOVA) followed by Tukey's multiple comparison test using GraphPad Prism 7 (version 7.05). A *p* value < 0.05 was considered statistically significant. Outliers were identified and removed using the ROUT test (GraphPad Software, Inc.), setting Q to 1.0%.

## 3. Results

### 3.1. Dual-Immunolabelling of SIRT3, SOD2, and GPX1 with *α*-Actin

**Figure 1:**
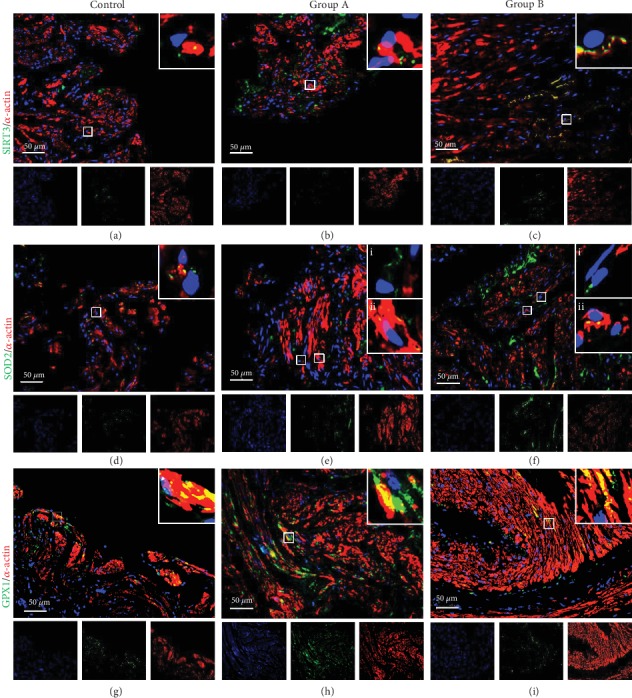
Representative images of dual-immunolabelling of SIRT3/*α*-actin, SOD2/*α*-actin or GPX1/*α*-actin in human penile tissue from controls, group A (≤2 CVDRF), and group B (≥3 CVDRF). SIRT3 (a–c), SOD2 (d–f), and GPX1 (g–i) were labelled green, *α*-actin red, and nuclei blue (DAPI). Colocalization of SIRT3, SOD2, or GPX1 with *α*-actin in smooth muscle was detected in yellow. Magnified areas are shown in the upper right boxes in images. Areas indicated by i and ii in SOD2/*α*-actin labelling in groups A and B (e, f) indicate SOD2 detection in non-muscle cells (green) and smooth muscle cells (yellow), respectively. The isolated blue, green, and red channels are shown for all images. CVDRF: cardiovascular disease risk factors; GPX1: glutathione peroxidase 1; SIRT3: Sirtuin 3; SOD2: superoxide dismutase-2.

Dual-immunolabelling was carried out to detect the expression of SIRT3, SOD2, or GPX1 (green) combined with *α*-actin (red), a specific marker of fusiform smooth muscle cells (Figure 1). While SIRT3 and SOD2 presented a dotted labelling compatible with their mitochondrial localization (Figures 1(a)–1(f)), GPX1 was detected in dots and also diffused in the cytoplasm (Figures 1(g)–1(i)). Nuclei were stained with DAPI (blue).

SIRT3 and *α*-actin coexpression (yellow) was rarely detected in tissues from all groups (Figures 1(a)–1(c)), but an increased labelling of SIRT3 was observed in group B (Figure 1(c)). Interestingly, our data evidenced a higher labelling of SOD2 in the smooth muscle in the control group (yellow) (Figure 1(d)). In the groups of patients with DM (groups A and B), scarce colocalization between SOD2 and *α*-actin was observed (Figures 1(e) and 1(f), magnified areas ii), but a green labelling corresponding to SOD2 expression in nonmuscular cells, such as fibroblasts or endothelium, was found (Figures 1(e) and 1(f), magnified areas i). Concerning GPX1 and *α*-actin, coexpression (yellow) was detected in all groups (Figures 1(g)–1(i)).

### 3.2. Western Blotting of NOX1, AMPK*α*, SIRT1, PGC-1*α*, SIRT3, SOD2, and GPX1*α*

**Figure 2:**
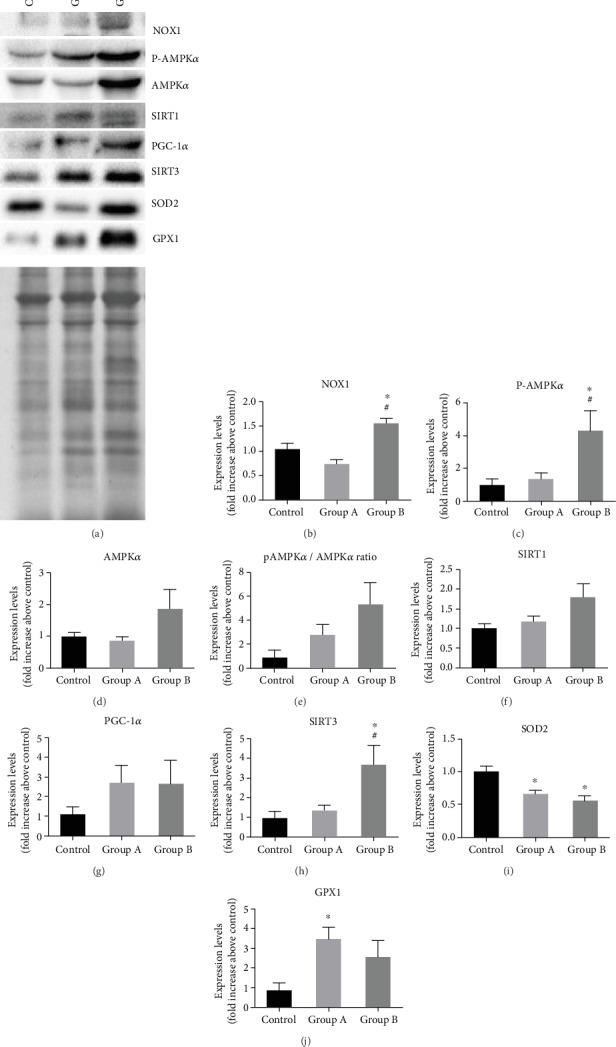
Western blotting semiquantification of NOX1, phospho-AMPK*α*, AMPK*α*, phospho-AMPK*α*/AMPK*α* ratio, SIRT1, PGC-1*α*, SIRT3, SOD2, and GPX1 levels. (a) Representative blots of each studied protein and respective representative Ponceau S protein staining. (b–j) Graphic representation of densitometric quantification of each band relative to Ponceau S staining (*n* = 4 − 6 per group). AMPK: AMP-activated protein kinase; GPX1: glutathione peroxidase 1; NOX: NADPH oxidases; PGC-1*α*: peroxisome proliferator-activated receptor *γ* (PPAR*γ*) coactivator-1*α*; SIRT1: Sirtuin 1; SIRT3: Sirtuin 3; SOD2: superoxide dismutase-2; ^∗^*p* < 0.05 relative to control; ^#^*p* < 0.05 relative to group A. Error bars represent the standard error of the mean.

To elucidate if the exposure to CVDRF deregulates AMPK*α*/SIRT1-PGC-1*α*-SIRT3 pathway, the expression levels of the intervenient proteins were semiquantified by western blotting. Bands with the expected molecular weight were identified for each studied protein: NOX1 65-68 kDa, AMPK*α* 62 kDa, SIRT1 140 kDa, PGC-1*α* 105 kDa, SIRT3 28 kDa, SOD2 25 kDa, and GPX1 22 kDa. Representative blots selected per group and the correspondent Ponceau S staining were shown in Figure 2(a). The quantification of expression of each protein was shown in the correspondent graphic (Figures 2(b)–2(j)).

NOX1 levels were increased in the CC samples of individuals belonging to group B, with a significant increment relatively to controls (*p* = 0.008) and group A (*p* = 0.0001). However, no difference was found between group A and control (*p* = 0.086).

Regarding AMPK*α* expression, both the phosphorylated and total forms were semiquantified. A significant increase in phospho-AMPK*α*, but not of total AMPK*α*, was found in group B relative to controls (*p* = 0.028) and group A (*p* = 0.038). However, the phospho-AMPK*α*/total AMPK*α* ratio only presented an increasing tendency in group B comparative with the others (group B vs. group A, *p* = 0.314, or controls, *p* = 0.072). While SIRT1 expression was equivalent in control and group A (*p* = 0.787), an almost significant increase was found in group B relative to controls (*p* = 0.050) and group A (*p* = 0.099).

Also, a 3-fold increment in PGC-1*α* was observed in CVDRF groups compared with controls, but owning to the inter-individual variation in groups, no statistical differences were found among them (control vs. group A (*p* = 0.451) or group B (*p* = 0.532)). SIRT3 expression instead was significantly increased in group B, when compared with control (*p* = 0.018) and group A (*p* = 0.0254). SIRT3 expression did not vary between group A and controls (*p* = 0.858). GPX1 expression was increased in groups with CVDRF compared with controls, but only between control and group A a significant difference (*p* = 0.032) was reached. Interestingly, SOD2 expression was found to decrease in group A (*p* = 0.004) and group B (*p* = 0.038) comparatively with the control group.

### 3.3. miR-421, miR-34a, miR-200a, and miR-206 Quantification by Real-Time PCR

**Figure 3:**
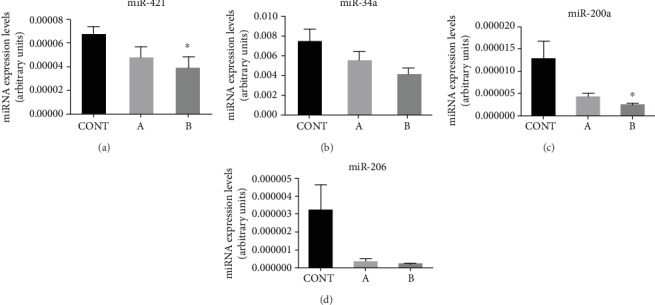
miR-421, miR-34a, miR-200a, and miR-206 level quantification by real-time polymerase chain reaction in human penile tissue sections. The formula 2^(CT RNU1A–CT RNx)^ was employed. ^∗^*p* < 0.05 relative to control. Error bars represent the standard error of the mean.

Quantification of miRNA levels in the human penile fragments was performed by real-time PCR (Figure 3). While miR-421 was found to significantly decrease in group B relative to controls (*p* = 0.043), no significant difference was achieved when comparing controls to group A (*p* = 0.219) or groups A and B (0.771). Additionally, miR-34a levels showed a decreasing tendency in groups of CVDRF patients comparative with controls without statistical significance; a probability *t*-test of *p* = 0.097 was found when comparing control with group B. miR-200a quantification by real-time PCR showed an overt decrease in groups A and B opposed to the control group (*p* = 0.084 and *p* = 0.046, respectively). Meanwhile, miR-206 levels tended to decrease in groups of CVDRF patients, but no statistical significance was obtained (control vs. group A (*p* = 0.121) or group B (*p* = 0.123)).

## 4. Discussion

DM induces noticeable oxidative modifications in cells, which in extension could be equivalent to those induced by the combination of two other CVDRF (smoking, hypertension, dyslipidaemia, and obesity) [42]. The risk factors associated to DM only explain about 25% of the additional oxidative burden in cardiovascular disease [42]. Hence, in the present study, the samples were firstly selected according to the presence of DM in patients and secondly according to the number of extra CVDRF, to identify the relative burden of DM and of the additional risk factors in oxidative damage response.

The prooxidative effects of hyperglycaemic states have been associated with NADPH oxidases activity increment [43], which agrees with our data, considering that a significant increment in NOX1 expression in group B, comparative to controls (*p* = 0.008), was found. The extra DM risk factors presented by the individuals of group B apparently have a relevant contribution to the NOX1 upregulation because relatively to group A, these patients also present higher levels of NOX1 (*p* = 0.0001).

In contrast with previous studies [24–27], we found SIRT3 expression to increase in the presence of CVDRF, considering the 3.6-fold increment observed in the patients exposed to a higher number of metabolic risk factors relative to controls (*p* = 0.018). This was an unexpected finding, taking into account that a decay of the levels of this enzyme, but not of the respective mRNA, was found in the CC of old patients with metabolic syndrome compared with the healthy counterparts [27]. We can speculate that middle-aged individuals, as those included in the current study, present cellular mechanisms able to mitigate SIRT3 degradation, possibly by preservation of functional mitochondria [44]. This in part could own to the availability of high levels of melatonin, a pineal hormone that confers a strong antioxidative protection of mitochondria and markedly decays along ageing [45]. The levels of SIRT3 protein also depend on mechanisms of silencing/degradation of the respective mRNA, for which the miRNA-421 has an important contribution. Cheng et al. reported miRNA-421 to negatively regulate SIRT3 by suppressing its translation and FOXO3 phosphorylation [36]. The higher levels of SIRT3 observed in group B agree with the low levels of miRNA-421 found in this group. In short, we can consider that in the middle-aged patients with multiple CVDRF, the higher levels of SIRT3 comparatively with those found in controls constitute a compensatory mechanism of defence against oxidative damage that could fail in older individuals [27].

Overexpression of SIRT3 has been demonstrated to increase SOD2 levels and activity, to defeat ROS-mediated damage through FOXO3 activation and ensuing gene expression or direct deacetylation of SOD2 [19, 21–23]. Interestingly, SOD2 was found to decrease in both groups of patients with CVDRF relatively to controls. Considering that in the individuals of group B an increment of SIRT3 and a correspondent decrement in miR-421 was observed, we could expect that the SIRT3/FOXO3 signaling pathway would be activated, which should result in FOXO3 phosphorylation and increased SOD2 expression [36]. Taking into account that in this study only the expression, but not the activity, of SIRT3 was measured, we cannot exclude that the downregulation of SOD2 could be strictly related with the activity of SIRT3. Additional studies to evaluate SIRT3 activity will be necessary to support this hypothesis.

The SOD2 downregulation in patients with CVDRF is partially in agreement with the findings of Chen et al. that reported a significant decline of SOD2 in diabetic mice aorta [46]. In the same study, however, a preserved expression of SOD2 was found in transgenic mice that overexpress SIRT1. The cross-talk between SIRT1 and SOD2 is yet to be determined, despite some studies reported a relationship between them through FOXO family (FOXO1 and 3a). Actually, Zhang et al. considered the SIRT1/FOXO/SOD2 pathway a major defence mechanism against ROS overproduction [47]. Our data did not show differences in the expression of SIRT1 among groups. Only an increasing trend that almost achieved a statistical significance was observed in group B, when compared with controls (*p* = 0.05). This finding opposes to previous studies that evidenced SIRT1 expression to be constitutively depressed in metabolic diseases like obesity and DM and in aged animals, while upregulated in starvation states [48–50]. However, it is in line with the previous study in the CC of aged patients that did not demonstrate differences among healthy individuals of several ages and aged with metabolic syndrome [27].

With the intent to clarify the regulatory mechanisms involved in SIRT1 expression, two miRNAs were studied: miR-34a and miR-200a. Actually, miR-34a-5p was found to directly target SIRT1 mRNA disrupting its pathway [34, 35]. Also, miR-34a was identified as the most upregulated miRNA in obesity conditions [34]. In our study, a decreasing tendency represented by a 54% reduction in miR-34a levels that almost reached statistical significance was observed between controls and group B (*p* = 0.097). This finding correlates with the increasing trend of SIRT1 found in group B. An additional support to this result is the significant downregulation in miR-200a that also targets SIRT1 mRNA, found in the patients with CVDRF, group B. Pan and colleagues reported, in contrary, a miR-200a upregulation in CC of rats with age-related erectile dysfunction associated with a decrement in SIRT1 [33]. But, differences along ageing could be expected as observed for SIRT3, because ageing is a multifactorial process that leads to progressive accumulation of defects that disturb the function of tissues, organs, and organisms. Regarding CC, ageing was demonstrated to be the single most significant risk factor for ED [51].

Additionally, we studied miR-206, a miRNA which levels highly relate with ED, and a decreasing tendency was observed in patients of group B; still no statistical differences were stablished. Nonetheless, despite the equivalent variation observed for miR-200a and miR-206, our findings disagree with data from Bai et al. and GamalEl et al. that reported an upregulation of both miR-200a and miR-206 in the CC of rats with obesity-related ED and in patients with nonmetabolic venoocclusive ED, respectively [30, 31].

Besides SOD2, GPX1 is an important intracellular antioxidant enzyme, in which expression and activity depend on multiple signaling pathways, including SIRT3, PGC-1*α*, and AMPK [52, 53]. Also, modulation of GPX1 in the skeletal muscle of the mouse is suggested to be mediated by nuclear factor *κ*B (NF*κ*B) [54] and p53 transcription factors, both regulated through SIRT1-catalysed deacetylation [55]. Interestingly, GPX1 has been associated with contradictory mechanisms in DM [10]; while some studies support a protective function for GPX1 in the preservation of pancreatic *β*-cells islets and hyperglycaemia reduction [56], others indicate that high levels of GPX1 in muscle skeletal cells contribute to insulin resistance [57]. In our study, GPX1 was found to significantly increase in the human CC samples from individuals with CVDRF, suggesting a compensatory defence mechanism against ROS overproduction supported by the rise in SIRT3. The increment of GPX1 relatively to SOD2 levels also suggests that the produced ROS could be efficiently degraded to innocuous products, owning to the sequential conversion to H2O2 by SOD2 and ultimately to H2O by GPX1. However, considering the controversy mentioned above, a deleterious effect of the increment in GPX1 on the muscle cells and on insulin regulation could not be excluded.

In line with the upregulation in SIRT3, an increment in phospho-AMPK*α* was found in group B. But we did not find a significant difference neither in total AMPK*α* and phospho-AMPK*α*/total AMPK*α* ratio nor in PGC-1*α* expression among groups, yet an increment tendency was observed in patients with metabolic diseases. Taken together, the increasing trends in total AMPK*α*, SIRT1, and PGC-1*α* and the increment of phospho-AMPK*α* and SIRT3 observed in patients of group B indicate a compensatory mechanism for the oxidative burden associated with the increase in NOX1.

The current study presents some limitations that may explain the absence of statistical differences in the levels of the studied proteins among the groups of patients. The main reason is the low number of patients in each group. In addition, the CC samples were obtained in programmed surgeries or during organ harvesting for transplants, which implies that unknown putative confounders such as exercise practice, diet, uncertain alcohol, or abusive substance use and prescription of drugs that interfere with AMPK/SIRT1-PGC-1*α*-SIRT3 pathway, such as metformin that activates AMPK [58], may differ among patients of the same group. Differences in lifestyle could affect the oxidative burden and the cellular responses to oxidative damage. Third, the CVDRF in patients were established as a qualitative attribute, which implies that the grade of the exposure to CVDRF was not entirely quantified in each individual. For further studies, it would be interesting to detail the metabolic profile and quantify parameters such as the obesity index, HbA1c, and levels of oxidised LDL and oxidised proteins in blood. Also, additional clinical information, such as time of diagnosis, medication protocol, presence of complications associated with DM and hypertension, cardiac resistance test, and lifestyle habits through a questionnaire could help refining our data.

## 5. Conclusions

In summary, the AMPK/SIRT1-PGC-1*α*-SIRT3 pathway and miR-421, miR-34a, miR-200a, and miR-206 were studied for the first time in penile tissue from diabetic middle-aged men, demonstrating that CVDRF additional to DM have a major impact in the response to oxidative damage. The accumulation of multiple CVDRF resulted in AMPK/SIRT1-PGC-1*α*-SIRT3 pathway activation, as a protective mechanism. The overall increment of SIRT3 expression, however, was not reflected in the expression of SOD2, considering that a downregulation of this enzyme was found. Hence, further analysis will be necessary to identify the regulatory mechanisms behind SOD2 and its implications in the response to clinically used drugs.

## Data Availability

Data employed in the conception and writing of this manuscript are available on Pubmed or Scopus. Any previous paper of the group not accessible could be requested to us.
